# Cyst Nematode Infection Elicits Alteration in the Level of Reactive Nitrogen Species, Protein *S*-Nitrosylation and Nitration, and Nitrosoglutathione Reductase in *Arabidopsis thaliana* Roots

**DOI:** 10.3390/antiox9090795

**Published:** 2020-08-26

**Authors:** Mateusz Labudda, Elżbieta Różańska, Marta Gietler, Justyna Fidler, Ewa Muszyńska, Beata Prabucka, Iwona Morkunas

**Affiliations:** 1 Department of Biochemistry and Microbiology, Institute of Biology, Warsaw University of Life Sciences-SGGW, Nowoursynowska 159, 02-776 Warsaw, Poland; marta_gietler@sggw.edu.pl (M.G.); justyna_fidler@sggw.edu.pl (J.F.); beata_prabucka@sggw.edu.pl (B.P.); 2 Department of Botany, Institute of Biology, Warsaw University of Life Sciences-SGGW, Nowoursynowska 159, 02-776 Warsaw, Poland; elzbieta_rozanska@sggw.edu.pl (E.R.); ewa_muszynska@sggw.edu.pl (E.M.); 3 Department of Plant Physiology, Poznań University of Life Sciences, Wołyńska 35, 60-637 Poznań, Poland; iwona.morkunas@mail.up.poznan.pl

**Keywords:** glutathione peroxidase, glutathione reductase, *Heterodera schachtii*, nitric oxide, nitrosative stress, non-symbiotic hemoglobin, oxidative stress, peroxynitrite, redox balance, *S*-nitrosothiol

## Abstract

Reactive nitrogen species (RNS) are redox molecules important for plant defense against pathogens. The aim of the study was to determine whether the infection by the beet cyst nematode *Heterodera schachtii* disrupts RNS balance in *Arabidopsis thaliana* roots. For this purpose, measurements of nitric oxide (NO), peroxynitrite (ONOO^−^), protein *S*-nitrosylation and nitration, and nitrosoglutathione reductase (GSNOR) in *A. thaliana* roots from 1 day to 15 days post-inoculation (dpi) were performed. The cyst nematode infection caused generation of NO and ONOO^−^ in the infected roots. These changes were accompanied by an expansion of *S*-nitrosylated and nitrated proteins. The enzyme activity of GSNOR was decreased at 3 and 15 dpi and increased at 7 dpi in infected roots, whereas the *GSNOR1* transcript level was enhanced over the entire examination period. The protein content of GSNOR was increased in infected roots at 3 dpi and 7 dpi, but at 15 dpi, did not differ between uninfected and infected roots. The protein of GSNOR was detected in plastids, mitochondria, cytoplasm, as well as endoplasmic reticulum and cytoplasmic membranes. We postulate that RNS metabolism plays an important role in plant defense against the beet cyst nematode and helps the fine-tuning of the infected plants to stress sparked by phytoparasitic nematodes.

## 1. Introduction

Reactive nitrogen species (RNS), especially nitric oxide (NO), play vital roles as biochemical regulators of growth and development processes during plants’ life, and they are involved in signal transduction in plant cells under both optimal and stressful environmental conditions. Furthermore, NO is a key regulator of solute transport, autophagy, and programmed cell death [1]. Numerous studies have also shown the interplay between NO and other molecules involved in signal transduction during plant defense response to biotic stress factors [2,3,4,5]. In plant cells, NO is mainly produced in two various pathways [6]. One of them is based on non-enzymatic transformations of nitrites under low pH or in the presence of reducing agent, for example, reduced ascorbate [7]. Secondly, NO can be released enzymatically because of nitrite ions reduction catalyzed by nitrate (NIA) or nitrite (NIR) reductases [8]. Despite the fact that nitric oxide synthase (NOS)-like activity (arginine-dependent NO production) was observed in vascular plants, this issue is still debatable, because we still have not known an enzyme or an enzymatic complex responsible for the NO production in this way in vascular plants [9]. Apart from NO, the group of RNS also includes peroxynitrite (ONOO^−^)—a molecule formed when NO reacts with the superoxide anion. Although the physiological role of ONOO^−^ is still poorly understood in plants, it is suggested that it may be important signaling molecule during plant defense responses against pathogen attack [10,11].

Posttranslational modifications (PTMs) of proteins significantly widen the proteome variety, enhance or reduce functionality, and lead to rapid cellular responses, all at comparatively low energetic costs for the plant organism [12]. One of the all-important functions of RNS is PTM of protein structure by nitration or *S*-nitrosylation. Nitrogen dioxide (NO_2_), a secondary product of NO metabolism, can modify especially tyrosine (Tyr) residue in *ortho* position in the phenolic hydroxyl group of Tyr resulting in the formation of 3-nitro-tyrosine (3-NT), which is a mainly irreversible reaction [13]. Besides Tyr, nitration can also occur on tryptophan and histidine residues. The formation of 3-NT in proteins can also be caused by ONOO^−^, which has a strong nitration activity for Tyr residues. Therefore, the presence of 3-NT in plant proteins is considered as the well-established marker of nitrosative stress induced by uncontrolled RNS generation, as nitrated proteins usually lose their biological function [14]. What is more, NO may reversibly bind the -SH group of cysteinyl residues, leading to *S*-nitrosothiols (SNOs) production, including an intracellular NO reservoir *S*-nitrosoglutathione (GSNO) as well as *S*-nitrosylation of proteins [15]. Such protein *S*-nitrosylation is involved in responses to nitro-oxidative stress, described as interplay between RNS and reactive oxygen species (ROS) generated under harsh environmental conditions [14]. It has been shown that *S*-nitrosylated proteins can participate in cell signaling, including phosphorylation, ubiquitylation, acetylation and related modifications, palmitoylation, and alternative cysteine-based redox modifications [16].

*S*-nitrosoglutathione as an endogenous SNO in plant cells plays a critical role in NO signaling and is a source of bioavailable NO. The intracellular amount of GSNO is controlled by *S*-nitrosoglutathione reductase (GSNOR), which catalyzes the nicotinamide adenine dinucleotide (NADH)-dependent reduction of GSNO to glutathione disulfide (GSSG) and ammonia (NH_3_). In this way, GSNOR regulates the level of NO in plants [14]. Another important enzyme involved in the maintenance of redox balance during nitro-oxidative stress is glutathione reductase (GR). GR regenerates pool of reduced glutathione (GSH), which in turn can be used by GSH-dependent antioxidant enzymes (including glutathione peroxidase, GPx) or, through reaction with NO, GSNO can be produced [15,17]. In addition to the above described mechanisms, the conversion of NO to nitrate by non-symbiotic class 1 hemoglobin (Hb1) showing nicotinamide adenine dinucleotide phosphate (NADPH)-dependent dioxygenase activity is responsible for NO homeostasis and bioactivity in plant cells [18].

The beet cyst nematode, *Heterodera schachtii* Schmidt, is a biotrophic parasite infesting crop and wild plants as well as *Arabidopsis thaliana* (L.) Heynh, a model plant species used to investigate the laws governing and shaping the plant–cyst nematode interaction. The infective second-stage juvenile (J2) of *H. schachtii* colonizes host roots and induces development of multicellular syncytium, which becomes the only source of nutrients for the feeding nematode [19]. The *A. thaliana* root invasion by *H. schachtii* causes the rapid production of ROS initiated by the plasma membrane NADPH oxidase. ROS are essential to limit plant cell death in infested *A. thaliana* roots and they promote syncytium induction [20]. In turn, the knowledge about the NO role and metabolism in nematode-infected plants is still extremely limited and only tomato and root–knot nematode *Meloidogyne incognita* interactions were analyzed [21,22]. Melillo et al. [21] found a swift NO presence at 12 h after *M. incognita* infection in tomato roots, whereas Zhou et al. [22] pointed to enhanced transcript levels of NO biosynthetic (nitrate reductase) and signaling (*S*-nitrosoglutathione reductase) genes at 7 days after infection. Therefore, on the basis of the above described data and our recently published results presenting significant alterations in the activity of nitrate and nitrite reductases and nitrate/nitrite amounts in *A*. *thaliana* plants infected with *H. schachtii* [23], it would be supposed that the changes in nitrogen metabolism induced by cyst nematode infection may coexist with altered RNS metabolism in infected plants. Thus, the aim of this research was to study the effect of *H. schachtii* infection on RNS metabolism in *A. thaliana* roots. To achieve the assumed research goal, the gene transcript level, protein level, and enzymatic activity of GSNOR, as well as the presence of NO and ONOO^−^ and patterns of protein nitration and *S*-nitrosylation, were evaluated in cyst nematode infected roots of *A*. *thaliana* plants. Additionally, the non-symbiotic hemoglobin 1 (Hb1) transcript level was measured to identify the potential cellular regulator of NO metabolism during infection.

## 2. Materials and Methods

### 2.1. Plant Growth Condition and Cyst Nematode Inoculation

Ten seeds (surface decontaminated) of *Arabidopsis thaliana* (L.) Heynh. ecotype Columbia (Col-0) were put into a Petri plate (9 cm in diameter). Plates were pre-filled with 0.2 × Knop medium (pH 6.4), which was coagulated with 0.7% agar. After fourteen days, *A. thaliana* roots were inoculated with 800–900 newly hatched juveniles of *Heterodera schachtii* Schmidt (J2s) per plate. J2s hatched from eggs in cysts, which were collected from *Sinapis alba* ‘Albatros’ root sterile agar culture. Parafilm-sealed plates were kept in a plant vegetation chamber MLR-350 (Sanyo, Tokyo, Japan) at 25 °C with a 16 h/8 h light/dark cycle under an irradiance (photosynthetic photon flux density) of 100  ±  25 μmol/m^2^/s [19]. Expansion of nematode infestation was observed during the first 3 days post-inoculation (dpi) using a stereo microscope; hence, infection time was accurately evaluated. Infected roots were collected for examinations after 3, 7, and 15 dpi. In addition, roots for RNS detection with confocal laser scanning microscope were also collected at 1 dpi. As a control, roots of uninfected plants were simultaneously sampled as well.

### 2.2. Detection of Reactive Nitrogen Species with Confocal Laser Scanning Microscopy

NO and ONOO^−^ generation in control and infected roots was observed under a Leica TCS SP5II inverted confocal laser scanning microscope (CLSM) (Leica Microsystems, Wetzlar, Germany) using a fluorescent dye, 4-amino-5-methylamino-20,70-difluorofluorescein diacetate (DAF-FM DA, Invitrogen-Molecular Probes, Paisley, UK) for NO or 3′-(*p*-aminophenyl) fluorescein (APF, Invitrogen) for ONOO^−^ according to the manufacturer’s instructions. To obtain a three-dimensional view, serial pictures sampled at various focal planes were processed by images stacking. The excitation/emission spectra were recorded at 488 nm/507–542 nm (for DAF-FM DA) or 488 nm/505–525 nm (for APF). In negative control samples, fluorescent probes were omitted (Figure S1).

### 2.3. Crude Extract Preparation

Roots (200 mg) of nematode infected and control plants were homogenized in a mortar with 1 mL of ice-cold extraction buffer (pH 7.8) containing 50 mM Tris-HCl, 2 mM 2-mercaptoethanol, 1 mM ethylenediaminetetraacetic acid (EDTA), 5% glycerol, 1 mM phenylmethylsulfonyl fluoride (PMSF), 5 mM MgCl_2_, and 2% polyvinylpyrrolidone (PVP). Homogenates were centrifuged (4 °C, 20 min, 16,000 relative centrifugal force (RCF)) and crude extracts were collected. The protein concentrations in crude extracts were measured using the standardized Bradford method and bovine serum albumin (BSA) (Sigma-Aldrich, Saint Louis, MO, USA) as a standard.

### 2.4. Profiling of S-Nitrosylated Proteins Patterns

The biotin switch procedure rested on the peculiar labelling of *S*-nitrosylated proteins and a biotin moiety on the *S*-nitrosylated cysteines was applied [24]. Proteins of crude root extracts (10 μg per lane, amounts standardized against α-tubulin level) were electrophoresed by the polyacrylamide gel electrophoresis (SDS-PAGE) and electrophoretically transferred to a nitrocellulose membrane (Mini-Protean electrophoresis system; Bio-Rad, Hercules, CA, USA). The electrophoresis conditions are described below in Section 2.8.2. in Materials and Methods. To verify the equability of protein amounts application to the gel, monoclonal mouse anti-α-tubulin antibodies (Sigma-Aldrich; 1:4000) and secondary horseradish peroxidase-conjugated goat antibodies against mouse IgG (Santa Cruz Biotechnology, Dallas, TX, USA; 1:5000) were used. The α-tubulin blots were visualized with the alcian blue method (Roche, Basel, Switzerland). The biotinylated proteins were also detected by immunoblotting. Briefly, after 2 h incubation at room temperature (RT) in 2.5% non-fat dried milk, the biotinylated proteins were revealed with anti-biotin-alkaline phosphatase antibodies produced in goat (Sigma-Aldrich; 1:500). The blots were visualized with a standard nitro-blue tetrazolium/5-bromo-4-chloro-3′-indolyphosphate (NBT/BCIP) solution containing 0.015% of BCIP and 0.03% of NBT in 10 mL of 0.1 M Tris-HCl buffer, pH 9.5, with 0.1 M NaCl, and 0.05 M MgCl_2_. To determine molecular weight (MW) of proteins, 2 µL of Spectra^TM^ Multicolor Broad Range Protein Marker (Thermo Scientific, Waltham, MA, USA) was used in all experiments.

### 2.5. Profiling of 3-Nitrotyrosine-Containing Proteins Patterns

Proteins of crude root extracts (10 μg per lane, amounts standardized against α-tubulin level as described above) were separated by SDS-PAGE and electrophoretically transferred to a nitrocellulose membrane. The 3-nitrotyrosines in proteins were detected by immunoblotting. After 2 h of RT incubation of membrane in 5% non-fat dried milk, the nitrated proteins were detected with anti-nitrotyrosine antibodies produced in rabbit (Sigma-Aldrich; 1:1000) and alkaline phosphatase-conjugated goat antibodies against rabbit IgG (Sigma-Aldrich; 1:20,000) as secondary antibodies. The blots were visualized with the NBT/BCIP solution as described previously. Blots with protein profiles (*S*-nitrosylated and 3-nitrotyrosines-containing) were digitalized with G:BOX EF2 (Syngene, Cambridge, UK) and the intensity of bands was quantified as % volume with free BioVision software (Vilber, Collégien, France). The average intensity of all bands was determined. All results were compared with control uninfected *A. thaliana* plants at 3 dpi, to which a value of 100% was assigned.

### 2.6. Glutathione Reductase (GR) Activity

The extract (10 μL) was mixed with an assay medium consisting of 50 mM Tris-HCl (pH 7.8), 250 μM NADPH, 1 mM EDTA, and 1 mM oxidized glutathione (GSSG) [19]. Reactions were carried out at 37 °C in a Nunc U-bottom 96-well plate (Thermo Scientific, Waltham, MA, USA) on a Varioskan LUX Multimode Microplate Reader (Thermo Scientific, Waltham, MA, USA) and the change in absorbance at 340 nm was monitored for 20 min with reads every 1 min. GR activity was expressed as mmol of oxidized NADPH per minute and gram of fresh weight (FW).

### 2.7. Glutathione Peroxidase (GPx) Activity

The extract (10 μL) was mixed with an assay medium consisting of 38 mM Tris-HCl (pH 8.8), 2.3 mM EDTA, 4.6 mM NaN_3_, 228.6 μM GSH, 9.5 mM Luperox^®^ (Sigma-Aldrich), and 476.2 μM Ellman’s reagent (5,5-dithiobis-2-nitrobenzoic acid, DTNB). GSH reacted with DTNB, producing 5-thio-2-nitrobenzoic acid (TNB), whose level was measured spectrophotometrically [25]. Reactions were carried out at 37 °C in a Nunc U-bottom 96-well plate on a Varioskan LUX Multimode Microplate Reader and the absorbance was measured at 412 nm. GPx activity was expressed in µmol of formed TNB per minute and gram FW.

### 2.8. Nitrosoglutathione Reductase (GSNOR): Enzymatic Activity and Protein Level

#### 2.8.1. Enzymatic Activity

The extract (10 μL) was mixed with an assay medium consisting of 50 mM Tris-HCl (pH 7.8), 200 μM NADH, 500 μM EDTA, and 600 μM GSNO (Sigma-Aldrich) [26]. Reactions were carried out at 37 °C in a Nunc U-bottom 96-well plate on a Varioskan LUX Multimode Microplate Reader. The change in absorbance at 340 nm was monitored for 20 min with reads every 1 min. GSNOR activity was expressed as µmol of oxidized NADH per minute and gram FW.

#### 2.8.2. Protein Gel Blot Analysis

Crude extracts containing 10 μg proteins were mixed with a sample buffer containing 126 mM Tris-HCl (pH 6.8), 20% glycerol, 4% sodium dodecyl sulphate (SDS), 10% 2-mercaptoethanol, and 0.004% bromophenol blue. After incubation at 95 °C for 5 min, protein samples were centrifuged (5 min, 16,000 RCF) and separated on 11% acrylamide gel with SDS in a 25 mM Tris, 192 mM glycine, and 0.1% SDS running buffer (pH 8.3) at 60 V for 15 min, followed by 1 h at a constant current of 25 mA per gel. SDS-PAGE separated proteins were transferred electrophoretically to a polyvinylidene fluoride (PVDF) membrane to detect the GSNOR protein level. After 1 h of blocking at RT with 5% non-fat dried milk, the membrane was incubated with anti-GSNOR polyclonal rabbit antibodies (Agrisera, Vännäs, Sweden; 1:1000) in 10 mL of phosphate-buffered saline (PBS) (pH 7.4) with 0.5% Tween 20. Alkaline phosphatase-conjugated goat antibodies against rabbit IgG (Sigma-Aldrich; 1:20,000) were used as the secondary antibodies. The blots were visualized using the NBT/BCIP solution, as described above. Blots were digitalized with G:BOX EF2 (Syngene) and bands’ intensity was quantified as % volume with BioVision software (Vilber). The results were compared with control uninfected *A. thaliana* plants at 3 dpi, to which a value of 100% was assigned. The original, uncropped, and unadjusted images supporting all blots can be found in Supplementary Materials.

#### 2.8.3. Transmission Electron Microscopy (TEM)

*A. thaliana* uninfected control roots and those containing syncytia at 3, 7, and 15 dpi were fixed in 2% paraformaldehyde and 2% glutaraldehyde in 10 mM PBS buffer pH 7.4 for 2 h. Samples were dehydrated, infiltrated, and embedded [27]. Immunogold labelling for TEM analyses was conducted on ultrathin sections (90 nm) obtained with a Leica UCT ultramicrotome (Leica Microsystems). Procedures were performed on nickel grid-mounted sections floating in drops [28]. Epoxy ultrathin sections were treated with 10% hydrogen peroxide (H_2_O_2_) for 10 min, and then they were pre-incubated in 10 mM PBS for 10 min. Unspecific binding sites were blocked by incubation with 4% BSA in 10 mM PBS for 1 h. Then, the incubation with anti-GSNOR polyclonal rabbit antibodies (Agrisera) diluted 1:100 with 10 mM PBS supplemented with 4% BSA for 4 h at RT was performed. Samples were washed in 10 mM PBS with 0.05% Tween 20 four times for 10 min and once in 10 mM PBS for 10 min. They were incubated for 1 h at RT in secondary goat anti-rabbit IgG conjugated with 15 nm colloidal gold particles (British BioCell International, Cardiff, UK) diluted 1:100 with 10 mM PBS supplemented with 1% BSA. The specimens were washed three times in 10 mM PBS with 0.05% Tween 20 and rinsed five times in distilled water. Grids were stained with 1.2% uranyl acetate solution in 70% methanol (Sigma-Aldrich) for 2 min and washed in distilled water five times for 2 min. In negative controls, primary antibodies were omitted (Figure S2). Samples were examined in an FEI 268D “Morgagni” (FEI Company, Hillsboro, OR, USA) transmission electron microscope operating at 80 kV. Images were collected with an SIS “Morada” (Olympus-SIS, Münster, Germany) digital camera. All obtained digital images were merged (if needed) using Photoshop CS6 (Adobe Systems Inc., San Jose, CA, USA) software (panorama tool), and adjusted using the same software by non-destructive tools (contrast, levels, and/or curves). All adjustments were made on the whole area of the image.

#### 2.8.4. Profiling of GSNOR Activity by Zymography in Native Polyacrylamide Gel

Each sample (protein content of 40 µg) was mixed with a nondenaturing, nonreducing sample buffer containing 125 mM Tris-HCl (pH 6.8), 20% glycerol, and 0.004% bromophenol blue. Native PAGE (Mini-Protean electrophoresis system) was run at 4 °C on 10% acrylamide gels in a 25 mM Tris-192 mM glycine running buffer (pH 8.3) at 60 V for 30 min followed by 5 h at a constant current of 25 mA per gel. After proteins’ separation by PAGE, gel was washed with sterile deionized water and then incubated in darkness for 15 min in a 100 mM Tris-HCl buffer (pH 7.8) containing 2 mM NADH. Next, gel was washed again with water and incubated in darkness for 15 min in a 100 mM Tris-HCl buffer (pH 7.8) containing 4 mM GSNO. Spots presenting GSNOR activity in the gel were detected after UV excitation [29]. Gels were digitalized with G:BOX EF2 (Syngene) and the intensity of spots was quantified as % volume with BioVision software (Vilber). The results were compared with control uninfected *A. thaliana* plants at 3 dpi, to which a value of 100% was assigned.

### 2.9. RNA Isolation and cDNA Synthesis

Total RNA was isolated from liquid N_2_-frozen uninfected and infected *A. thaliana* roots using the universal RNA purification column-based kit (EURx, Gdańsk, Poland) according to the manufacturer’s recommendations. RNA preparations were treated with RNase-free DNase I (EURx) on columns to genomic DNA digestion. The RNA content was measured spectrophotometrically, and its purity and integrity were controlled on a 1.2% agarose gel in presence of the SimplySafe dye (EURx). The original, uncropped, and unadjusted exemplary image supporting the RNA agarose gel electrophoresis can be found in Supplementary Materials. Isolated total RNA (2 μg) was reverse-transcribed using high-capacity cDNA reverse transcription kit with the MultiScribe Reverse Transcriptase and random primers (Applied Biosystems/Thermo Scientific).

### 2.10. Quantitative Real-Time PCR

Quantitative real-time PCR (qPCR) reactions were performed in Hard-Shell^®^ 96-well plates sealed with Microseal^®^ ‘B’ PCR films (Bio-Rad) on a CFX96 Touch™ Real-Time PCR Detection System with CFX Maestro™ software (Bio-Rad). qPCR mixtures contained 7.5 μL of PowerUP SYBR™ Green PCR Master Mix (Applied Biosystems/Thermo Scientific), 1.9 μL of sterile water, 200 nM primers, and 5 μL of 1:25 diluted first-strand cDNA template. We used the following qPCR conditions: AmpliTaq Gold^®^ polymerase (Applied Biosystems/Thermo Scientific) activation at 95 °C for 10 min, then 40 cycles consisting of denaturation at 95 °C for 15 s, and annealing/extension at 60 °C for 60 s with fluorescence reading. Sequences of intron-spanning primers were designed with the Universal Probe Library Assay Design Center Probe Finder software (Roche; http://www.roche-applied-science.com/). Designed sequences (5′–3′) were as follows: for *S-nitrosoglutathione reductase 1* (*GSNOR1*) gene (AT5G43940, gene ID number 834417), 97 bp amplicon size: ctttgtcacaccgacgctta and cactctcaacaatcccagca; *non-symbiotic hemoglobin* (*HB1*) gene (AT2G16060, gene ID number 816103), 100 bp amplicon size: ggaaagttacggtgagggaga and aatgcatacttggccacctc; *ubiquitin specific protease 22* (*UBP22*) gene (AT5G10790, gene ID number 830946), 121 bp amplicon size: cacaaggggatgttggaatcag and actcacatcctctcaccacttc. *UBP22* demonstrated a stable gene transcript level over the whole period of experiment, thus it was used as the endogenous reference gene.

### 2.11. Statistics

The results are shown as the arithmetic means ±SD from three independent biological replicates. The results were evaluated with two-way analysis of variance (ANOVA) (factors: infection and duration of infection). A post hoc Tukey’s test was implemented to determine significant differences between means at *p* < 0.05 (Statistica 13.3; StatSoft Inc., Tulsa, OK, USA). The gene transcript level of each gene was tested in three biological replicates and three technical repetitions. The specificity of amplified PCR products was verified by melting curve analysis. For statistical analysis, the calculation of reaction efficiency was performed using LinRegPCR software version 2020.0.0.0.3 (http://LinRegPCR.nl), whereas the relative gene expression levels and statistical significance of their differences were estimated by Relative Expression Software Tool 2009 (REST 2009) (http://rest.gene-quantification.info/) as described previously [30] and confirmed using Student’s *t*-test and Fisher’s *F*-test (Statistica 13.3; StatSoft Inc.).

## 3. Results

### 3.1. Generation and Localization of Reactive Nitrogen Species

**Figure 1:**
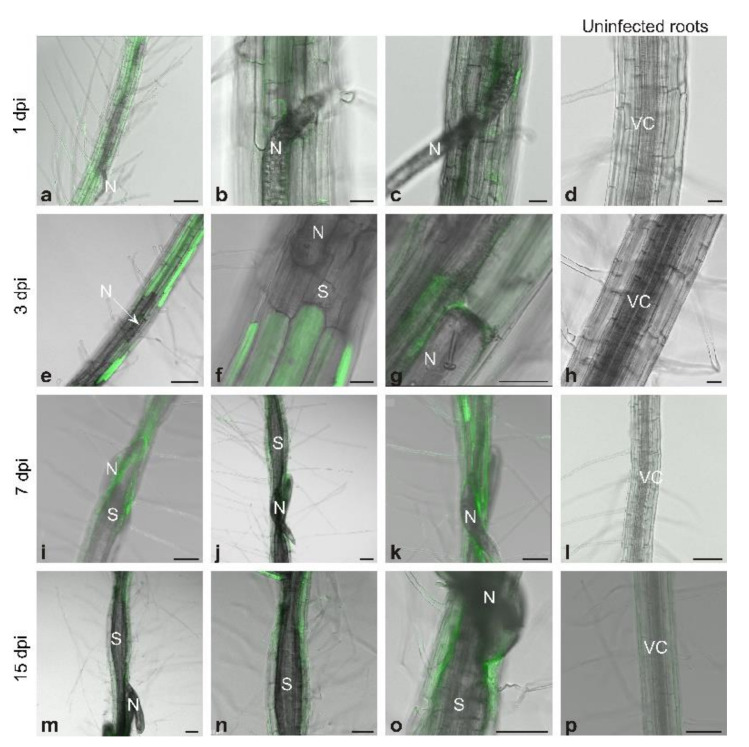
Nitric oxide detection in *Heterodera schachtii*-infected *Arabidopsis thaliana* roots at 1 dpi (**a**–**c**), 3 dpi (**e**–**g**), 7 dpi (**i**–**k**), and 15 dpi (**m**–**o**) and uninfected control roots (**d**,**h**,**l**,**p**). Abbreviations: dpi—days post-inoculation, N—nematode, S—syncytium, VC—vascular cylinder. Scale bars: 20 µm (**b**–**d**,**f**,**g**,**l**) and 100 µm (**a**,**e**,**h**–**k**,**m**–**p**).

The DAF-FM DA fluorescent probe was used to visualize the presence of NO in roots and in developing syncytia (Figure 1). At 1 dpi, NO was detected in root cells close to the J2 penetration zone, along the nematode migration road, as well as close to the nematode head (Figure 1a–c). At 3 dpi, the fluorescent signal in infected roots was localized in the vascular cylinder and cortex cells surrounding syncytium and close to the nematode head (Figure 1e–g). At 7 and 15 dpi, NO was still present near to the nematode head (Figure 1i,o), and it was faintly visible in vascular cylinder cells surrounding syncytia (Figure 1i,j,m,n). Moreover, it was almost undetectable inside syncytia (Figure 1i–k,m–o). In uninfected roots examined at days corresponding to the infection, specific fluorescence signals originating from NO were not detected using this applied analytic method (Figure 1d,h,l,p).

**Figure 2:**
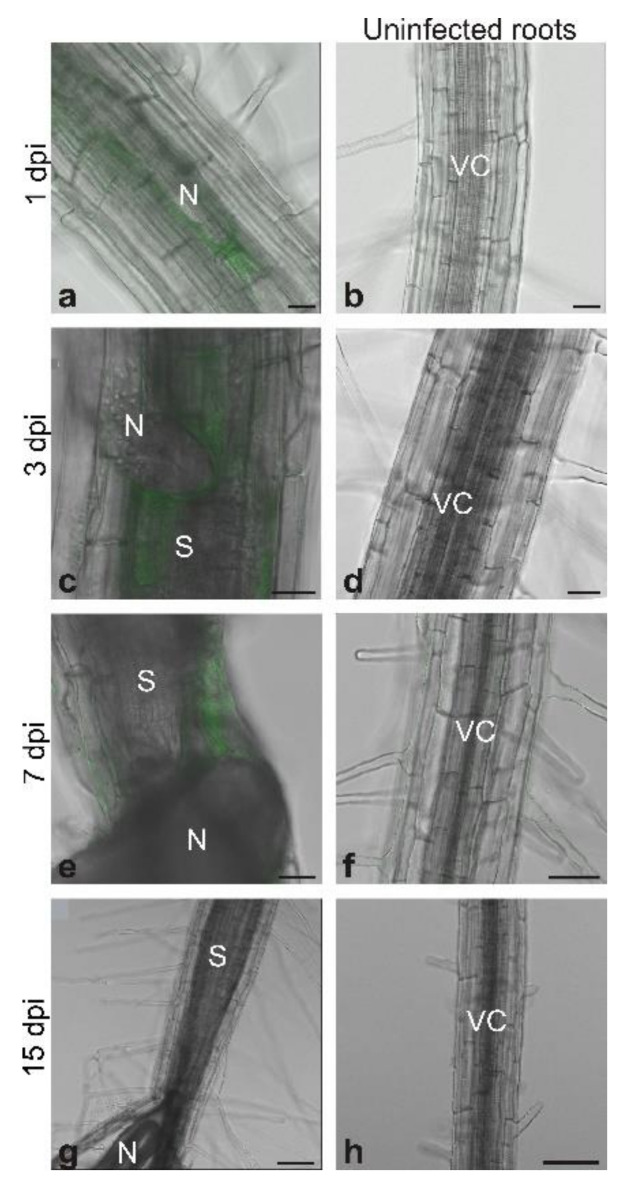
Peroxynitrite detection in *Heterodera schachtii*-infected *Arabidopsis thaliana* roots at 1 dpi (**a**), 3 dpi (**c**), 7 dpi (**e**), and 15 dpi (**g**) dpi and uninfected control roots (**b**,**d**,**f**,**h**). Abbreviations: dpi—days post-inoculation, N—nematode, S—syncytium, VC—vascular cylinder. Scale bars: 20 µm (**a**–**f**) and 100 µm (**g**,**h**).

Peroxynitrite was localized with APF fluorescent probe in the vascular cylinder cells near to the nematode head at 1, 3, and 7 dpi (Figure 2a,c,e). At the later stage of the cyst nematode infection (15 dpi), ONOO^−^ was undetected neither in the cells of the infected roots nor in the syncytia (Figure 2g). APF specific fluorescence signals were also not observed in cells of uninfected roots (Figure 2b,d,f,h).

### 3.2. Protein S-Nitrosylation and Nitration

**Figure 3:**
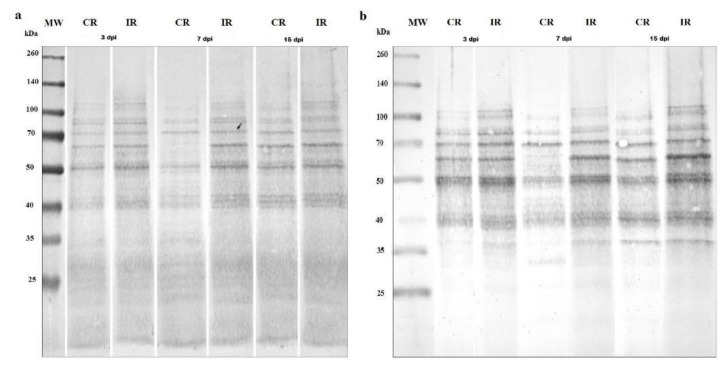
Patterns of *S*-nitrosylated (**a**) and nitrated (**b**) proteins in *Heterodera schachtii*-infected *Arabidopsis thaliana* roots at 3, 7, and 15 dpi. Equal amounts of proteins applied to the gel were standardized against α-tubulin level. Abbreviations: CR—control roots, dpi—days post-inoculation, IR—infected roots, MW—molecular weight.

During the response of *A. thaliana* roots to infection, the general protein *S*-nitrosylation level (estimated densitometrically) was increased by 22% at 3 dpi, 27% at 7 dpi, and 13% at 15 dpi in infected roots in comparison with control ones (Figure 3a). Moreover, *S*-nitrosylation increased with plant age regardless of the infection, reaching at 15 dpi a level 32% higher than at 3 dpi (Figure 3a). Not only the overall *S*-nitrosylation, but also patterns of posttranslationally modified protein changes in different dpi. In comparison with control plants, lower intensities of bands with MW 55 kDa–65 kDa and ~100 kDa were observed in infected roots at 7 dpi, but at the same time point, there were relatively high intensities of bands with MW ~70 kD, as well as 35 kDa and lower. In control roots at 15 dpi and in infected root samples at 3, 7, and 15 dpi, a high level of *S*-nitrosylation was noticed in proteins with MW ~60 kDa, ~50 kDa, and 45–40 kDa. In the comparison of infected roots at 3 dpi with infected roots collected at 7 and 15 dpi, there was a higher concentration of ~110 kDa and ~80 kDa *S*-nitrosylated proteins, and a lower concentration of ~45 kDa *S*-nitrosylated proteins (Figure 3a).

In control uninfected root samples, the level of protein nitration depended on plant age and was the lowest in plants at 7 dpi (Figure 3b). *A. thaliana* root infestation by *H*. *schachtii* increased the general nitration level by 44% at 3 dpi, 94% at 7 dpi, and 137% at 15 dpi in comparison with appropriate control plants (Figure 3b). Similarly to *S*-nitrosylation, not only the general protein nitration level, but also patterns of nitrated proteins differed between root samples. In control roots at 7 dpi, in comparison with other control root samples at 3 and 15 dpi, the intense nitration of proteins with MW ~70 kDa accompanied by the lack of nitration of proteins with MW ~60 kDa was observed. In turn, the intense nitration of proteins with MW ~37 kDa was determined both in control roots at 15 dpi and in infected roots at 3, 7, and 15 dpi. Additionally, in infected roots, the nitration of 90–110 kDa proteins appeared independently of the measurement time (Figure 3b).

### 3.3. GSNOR Protein Level and Enzyme Activity

**Figure 4:**
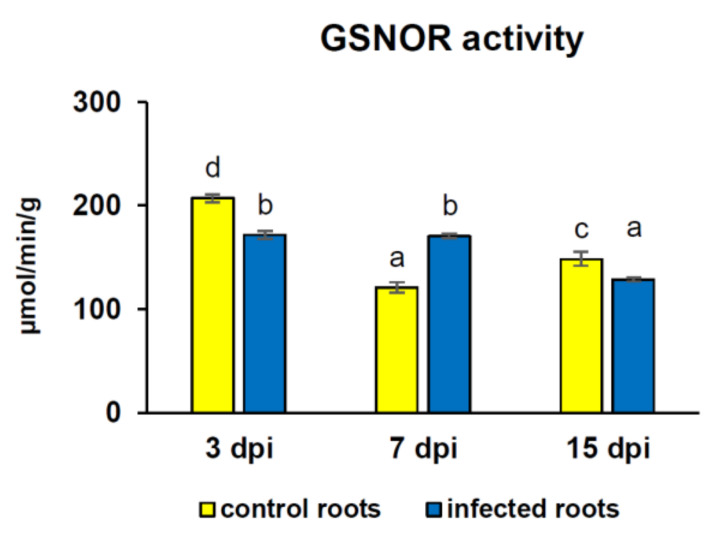
Nitrosoglutathione reductase (GSNOR) activity [µmol/min/g] in *Heterodera schachtii*-infected *Arabidopsis thaliana* roots at 3, 7, and 15 dpi. Data are presented as means ±SD. Different letters indicate means that are significantly different at *p* < 0.05 according to two-way analysis of variance and a post hoc Tukey’s test. Abbreviation: dpi—days post-inoculation.

**Figure 5:**
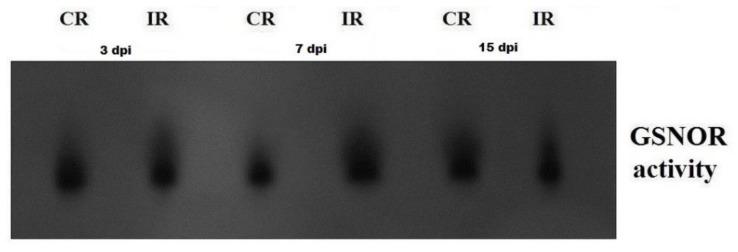
Zymography of nitrosoglutathione reductase (GSNOR) activity in *Heterodera schachtii*-infected *Arabidopsis thaliana* roots at 3, 7, and 15 dpi. Abbreviations: CR—control roots, dpi—days post-inoculation, IR—infected roots.

The GSNOR activity in roots depended on infection, duration of infection, and the combination of these two factors. The GSNOR activity was 1.2-fold lower in infected roots at 3 and 15 dpi than in control samples (Figure 4). At 7 dpi, GSNOR activity in roots was enhanced about 1.4-fold because of *H*. *schachtii* parasitism (Figure 4). Similarly, in-gel GSNOR activity was higher in infected roots than in corresponding controls at 7 dpi; however, at 15 dpi, it was decreased (Figure 5).

**Figure 6:**
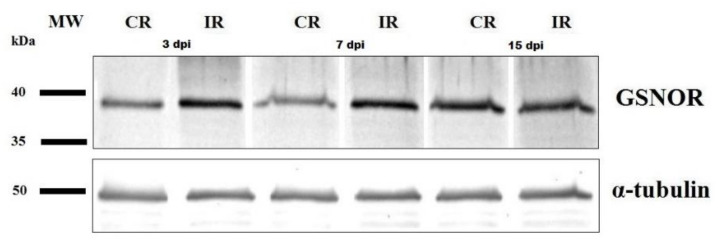
Nitrosoglutathione reductase (GSNOR) protein level in *Heterodera schachtii*-infected *Arabidopsis thaliana* roots at 3, 7, and 15 dpi. Equal amounts of proteins applied to the gel were standardized against α-tubulin level. Abbreviations: CR—control roots, dpi—days post-inoculation, IR—infected roots, MW—molecular weight.

Densitometric analysis of GSNOR protein bands revealed that, during *A. thaliana* response to infection, the protein content of GSNOR increased in infected roots by approximately 60% at 3 dpi and 90% at 7 dpi in comparison with control plants (Figure 6). However, the level of GSNOR protein in older plants was notably higher in control roots and there was no significant difference between uninfected and infected plants at 15 dpi (Figure 6).

**Figure 7:**
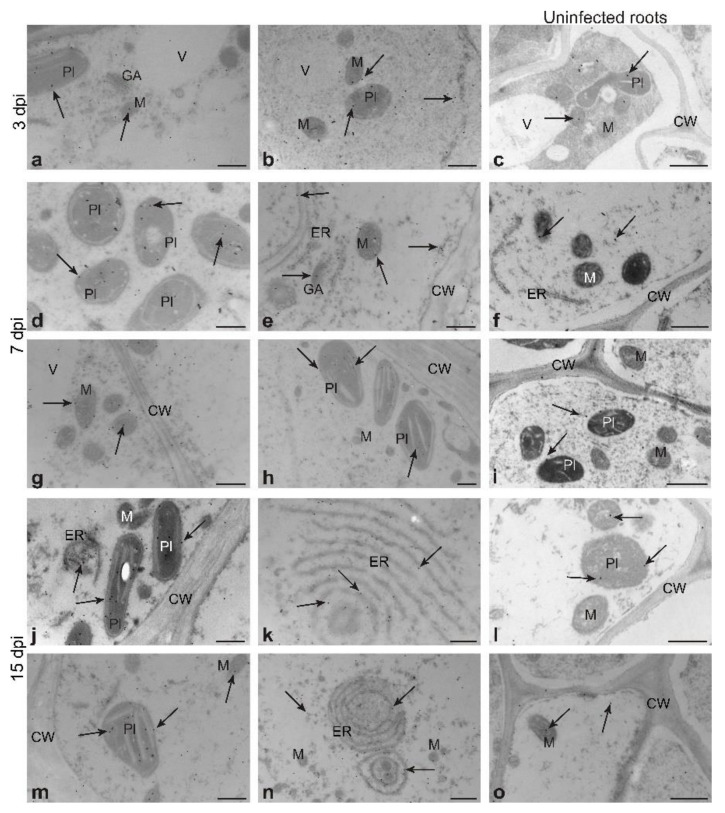
Localization of nitrosoglutathione reductase protein (GSNOR) by immunogold labelling and transmission electron microscopy in *Heterodera schachtii*-induced syncytia in *Arabidopsis thaliana* roots at 3 (**a**,**b**), 7 (**d**,**e**,**g**,**h**), and 15 (**j**,**k**,**m**,**n**) dpi and uninfected control roots (**c**,**f**,**i**,**l**,**o**). Abbreviations: CW—cell wall, dpi—days post-inoculation, ER—endoplasmic reticulum, GA—Golgi apparatus, M—mitochondrion, Pl—plastid, V—vacuole. Arrows indicate gold particles of GSNOR protein. Scale bars: 0.5 µm. Abbreviation: dpi—days post-inoculation.

To learn more about GSNOR during cyst nematode infection, we performed immunogold labelling at ultrastructural level. The presence of GSNOR protein was observed in cells of uninfected roots as well as in syncytial elements (Figure 7). At all examined time points of syncytium development, gold grains localized GSNOR protein predominantly in plastids (Figure 7a,d,h,j,m), mitochondria (Figure 7b,e,g,n), cytoplasm (Figure 7b,e), and endoplasmic reticulum membranes (Figure 7k,n), and sporadically in plasmalemma (Figure 7e). To a lesser extent, gold grains were observed in the same organelles of uninfected roots (Figure 7c,f,i,l,o).

### 3.4. Activity of Glutathione-Dependent Enzymes

**Figure 8:**
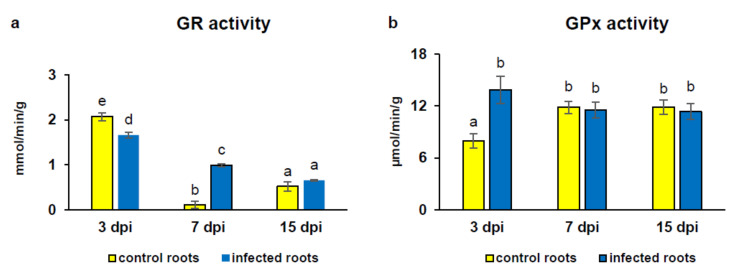
Glutathione reductase (GR) [mmol/min/g] (**a**) and glutathione peroxidase (GPx) [µmol/min/g] (**b**) activities in *Heterodera schachtii*-infected *Arabidopsis thaliana* roots at 3, 7, and 15 dpi. Data are presented as means ±SD. Different letters indicate means that are significantly different at *p* < 0.05 according to two-way analysis of variance and a post hoc Tukey’s test. Abbreviation: dpi—days post-inoculation.

The activity of GR in roots of *A*. *thaliana* depended on all considered factors: infection, duration of infection, and their combination. The GR activity was 1.2-fold lower in infected roots at 3 dpi and 9-fold higher at 7 dpi as a result of *H*. *schachtii* infection, whereas its activity at 15 days of experiment did not differ significantly between infected and uninfected roots (Figure 8a). In turn, the GPx activity depended on infection and its interaction with the duration of infection. The GPx activity presented an enhanced level (1.7-fold) in infected roots at 3 dpi in comparison with control ones and, at 7 and 15 days of experiment, did not vary significantly between infected and control roots (Figure 8b).

### 3.5. Alterations in Gene Transcript Level of GSNOR and HB1

**Figure 9:**
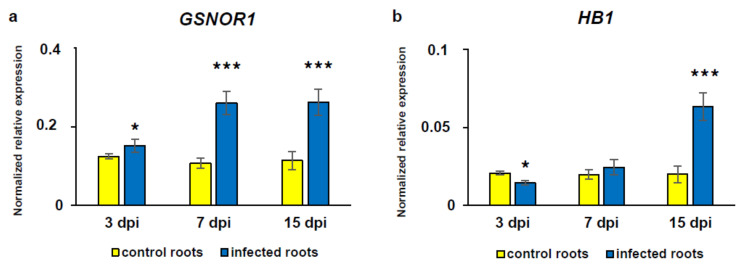
The analysis of changes in *Arabidopsis thaliana* nitrosoglutathione reductase (*GSNOR1*) (**a**) and non-symbiotic hemoglobin 1 (*Hb1*) (**b**) transcript levels during *Heterodera schachtii* infection at 3, 7, and 15 dpi. The relative gene expression levels and statistical significance of their differences were estimated by REST 2009 software and confirmed using Student’s *t*-test and Fisher’s *F*-test. The transcript level for each gene was normalized to the endogenous controls. Bars represent means ±SE (*n* = 3); asterisks indicate statistically significant differences in comparison with the control uninfected roots at *p* < 0.05 (*), *p* < 0.001 (***). Abbreviation: dpi—days post-inoculation.

The *GSNOR1* transcript level in infected roots at 3 dpi was slightly, but significantly increased (1.3-fold) in comparison with the uninfected ones, whereas at 7 and 15 dpi, the transcript level of this gene was about 2.4-fold higher than in respective controls (Figure 9a). Further, it was found that *HB1* transcript level was 2-fold lower in infected roots at 3 dpi and 3-fold higher at 15 dpi than in uninfected ones, while at 7 dpi, the *HB1* transcript level did not differ significantly between infected and control roots (Figure 9b).

## 4. Discussion

**Figure 10:**
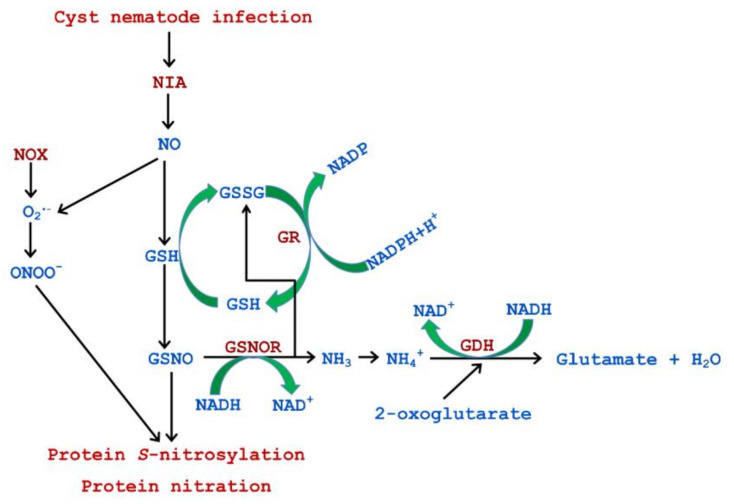
Simplified scheme of reactive nitrogen species metabolism and its proposed connections with glutathione, nitrosoglutathione (GSNO), as well as glutamate metabolism and protein *S*-nitrosylation and nitration in *Arabidopsis thaliana* roots infected with *Heterodera schachtii*. Nitric oxide molecules were produced by nitrate reductase (NIA) [23] and superoxide anion molecules by nicotinamide adenine dinucleotide phosphate (NADPH) oxidase (NOX) [20]. Ammonium ions (NH_4_^+^) formed because of the nitrosoglutathione reductase (GSNOR) activity were consumed by glutamate dehydrogenase (GDH), while glutathione disulphide (GSSG) produced by GSNOR was used by glutathione reductase (GR), which regenerated reduced glutathione (GSH) [23].

Reactive nitrogen species may be considered as a double-edged sword on the plant and cyst nematode battlefield. On the one hand, they can lead to the loss of biological properties of proteins through nitration of tyrosines [14], but on the other hand, RNS can play an important signaling role when plant responds to infection [1]. Here, for the first time, we have pointed out the significance of RNS metabolism during *A. thaliana* roots infection with *H. schachtii* (Figure 10). It is now clear that RNS are produced by root cells in response to the cyst nematode attack and parasitism.

One of the regulatory targets of NO in cells is the mitogen protein kinases (MPKs) cascade, which is a pivotal component of protein phosphorylation cascades transducing signals during plant responses against pathogens [31]. It has been proven that MPK3, MPK6, and MPK phosphatase AP2C1 were important regulators during *A. thaliana* infection with *H. schachtii*; their joint action led to timely initiation of attacked plant defense [32]. Interestingly, the changes observed by these authors were particularly intensely noticeable during the early stages of infection, when J2s pierced the rhizodermis, migrated inside roots, and induced syncytia. These observations reflect the intense DAF-FM DA probe signal observed by us, showing the abundant content of NO in infested *A. thaliana* roots at 1 and 3 dpi. These may indirectly suggest that NO participates in the regulation of the MPKs cascade. Our inference is also confirmed by the results from another pathosystem based on *Glycine max* infection with *Heterodera glycines* [33]. It was shown that MPKs, including soybean homologs of *A. thaliana* MPK3 and MPK6 described above, had a defensive role during the *G. max* response to *H. glycines* infection. Interestingly, in addition to the argumentation presented above, another face of the NO–MPKs interaction should be considered, especially taking into account the results presented earlier by our team in the article on the metabolism of nitrogen in *H. schachtii*-infected *A. thaliana* plants [23]. Siddique et al. [20] showed elevated H_2_O_2_ production in *A. thaliana* roots as a result of the *H. schachtii* infestation. Furthermore, Wang et al. [34] proved that H_2_O_2_-mediated activation of MPK6 in roots of *A. thaliana* led to the phosphorylation of nitrate reductase 2 (NIA2) by MPK6, resulting in increased nitrate reductase enzyme activity and NO liberation. It cannot be overlooked that enhanced transcript level of the NIA2 gene was responsible for the increased enzyme activity of nitrate reductase in *H. schachtii*-infected *A. thaliana* roots at 3 dpi [23]. These data indicate that, at least to some extent observed by us herein, the intense NO burst in the cyst nematode infested *A. thaliana* roots at the early stage of infection resulted from higher (3-fold) nitrate reductase activity in infected roots than in control samples (Figure 10) [23]. Non-enzymatic production of NO from nitrites in the presence of reduced ascorbate [7] cannot be excluded because of a decrease in nitrite content at 3 dpi [23] and an increase in reduced ascorbate content in *H. schachtii*-infected *A. thaliana* roots observed [35]. On the contrary, Melillo et al. [21] suggested that the arginine-dependent NO production (NOS-like) rather than nitrate reductase was the main source of NO in the roots of tomato plants infected with *M. incognita*. In our case, the arginine-dependent pathway does not occur because the arginine content in *H. schachtii*-infected *A. thaliana* roots was similar to that in uninfected ones [19], suggesting a different response of the host plant to the infection with the root-knot and cyst nematode.

The results of our experiments have shown that *H*. *schachtii*-infected *A*. *thaliana* roots showed elevated levels of ONOO^−^ from 1 to 7 dpi. ONOO^−^ is formed when NO reacts with the superoxide anion [11]. Taking into account that Siddique et al. [20] proved the production of superoxide anions (by the plasma membrane NADPH oxidase) as a result of *H. schachtii* infection, which in turn reacted with NO, there is no doubt that ONOO^−^ participated in the defense reaction of *A*. *thaliana* roots against parasitic nematode, because it may easily migrate through cell membranes and interact with biotargets (Figure 10) [11]. Besides, its involvement in defense responses of potato plants against *Phytophthora infestans* was shown [10].

As a result of the primary stress, in our case, the cyst nematode infestation, the nitro-oxidative stress can be secondarily induced. Thus, a plethora of produced RNS can nitrate tyrosines in proteins to 3-nitro-tyrosines (3-NTs), whose presence is a widely known marker of nitro-oxidative stress in plants [36]. We noticed that the content of 3-NTs increased linearly in infected roots from 3 to 15 dpi, indicating the gradual increase of the nitro-oxidative stress intensity in *H. schachtii*-infected *A. thaliana* roots. Furthermore, according to Mata-Pérez et al. [36], three different physiological consequences can appear depending on the intensity level of nitro-oxidative stress. At the beginning of the cyst nematode infection (3 dpi), tyrosine nitration in proteins can cause a beneficial modification of physiological processes in order to promote the fine-tuning of the infected plants against nitro-oxidative stress induced by parasitic cyst nematodes. When the intensity of the infection-induced nitro-oxidative stress becomes increased (7 dpi), the dysregulation of plant metabolic and/or physiological functions can be exerted. Finally, under prolonged stress condition (15 dpi), nitro-oxidative stress can provoke accumulation and aggregation of proteins containing 3-NTs and their denaturation, and these symptoms are typical for the senescence of roots [37]. We mentioned above the non-enzymatic tyrosine nitration by NO_2_ and ONOO^−^. However, in mammals, it has been known that myeloperoxidase, a major heme enzyme of neutrophils, monocytes, and some populations of macrophages, can mediate enzymatic tyrosine nitration in the presence of H_2_O_2_ and nitrite ions during peritoneal inflammation induced by *Klebsiella pneumoniae* infection [38]. As the lifetime of ONOO^−^ is truly short at a physiological pH, it is virtually impossible to control the reaction in the cells. In contrast, the nitration reaction catalyzed by peroxidases can be controllable. In relation with pathogen response, ROS production is generally observed in plants and animals. It is important to be reminded that H_2_O_2_ is required for the enzymatic nitration. In plants, guaiacol peroxidase such as horseradish peroxidase may mediate not only protein nitration, but also the nitration of phenolic compounds [39]. Both of these aspects are extremely interesting and poorly researched in the context of plant stress response, thus they open and mark new research paths.

As *S*-nitrosylation is a reversible reaction, it causes rather different physiological effects compared with nitration. This type of PTM participates in nitro-oxidative stress responses via, among others, the regulation of antioxidant defense enzymes’ activity, such as ascorbate peroxidase, monodehydroascrobate reductase, dehydroascorbate reductase, as well as GSNOR [36]. It was noted that the general protein *S*-nitrosylation level was enhanced in infected roots of *A*. *thaliana* (from 3 to 15 dpi) in comparison with control uninfected ones. These observations clearly suggest that regulatory processes dependent on *S*-nitrosylation of proteins take place during plant host reaction to the attack of cyst nematodes. However, to determine which of the enzymes and/or non-enzymatic proteins are regulated by this PTM, future proteomic studies are needed.

GSNOR controls the amount of GSNO, so it participates in the governing of NO activity and metabolism. Changes in GSNOR on both the transcriptional and post-translational level can orchestrate NO signaling pathways in plants [15]. It was observed that GSNOR protein level and enzymatic activity did not always go hand in hand with the *GSNOR1* gene transcript level. This observation strongly suggests that regulation of GSNOR activity during cyst nematode infection is intricate and cannot be seen only as simply transferring higher gene expression into higher enzymatic activity, and, for example, the post-translational modifications of GSNOR protein are possible. At the early stages of infection (3 dpi), the *GSNOR1* transcript level was slightly, but significantly increased, together with the noticeable higher GSNOR protein level in infected roots, but surprisingly, this enzyme activity was significantly inhibited. A similar trend was observed at 15 dpi in infected roots, however, there was no significant difference in GSNOR protein between uninfected and infected roots. Zhan et al. [40] showed that GSNOR *S*-nitrosylation at the highly conserved cysteine-10 resulted in protein destabilization, which induced conformational modifications and facilitated selective degradation via autophagy. Thus, as noticed by us, partly inhibited GSNOR activity at 3 and 15 dpi could be a consequence of GSNOR *S*-nitrosylation. Moreover, GSNOR was proved to be a long-lived protein with a half-life time longer than 24 h [40]. Therefore, it cannot be ruled out that *S*-nitrosylated GSNOR might be degraded in the autophagosome between sampling points, as no reduction in GSNOR protein level was observed at the tested dpi. This finding is in accordance with our previous study on Ca^2+^-dependent cysteine proteases (calpains), which are known to participate in long-lived protein degradation through the autophagic process in mammalian cells [41]. It was found that the activity of calpains in *A*. *thaliana* roots can be highly stimulated as a result of cyst nematode infection [42]. Summarizing, the inhibition of GSNOR activity accompanied by an enhancement in the *S*-nitrosothiols accumulation can express in enhanced plant immunity against pathogens [15].

In contrast to 3 and 15 dpi, when reduced catabolism of GSNO in infected roots was noticed at 7 dpi, the GSNOR activity was enhanced, indicating elevated GSNO breakdown. Thereby, the stimulation of the scavenging of the excess number of NO via GSH *S*-nitrosylation appeared, and consequently, uncontrolled effects of nitro-oxidative stress on functioning of plants under nematode infection were limited. This system seems to work effectively because it was noted that the activity of GR was induced in infected roots at 7 dpi, so the regeneration of GSH from GSSG produced by GSNOR occurred (Figure 10). What is more, unchanged activity of GPx using GSH in infected roots in comparison with controls was detected. Thus, further GSH molecules could also react with NO to form GSNO, which became a GSNOR substrate. It is impossible not to mention another GSNOR product, namely NH_3_. In plant cell, it is present as ammonium ions, which in turn are substrates for glutamate dehydrogenase (GDH), catalyzing the reversible reductive amination of 2-oxoglutarate to glutamate. This has also been previously shown by our team as the activity of GDH was significantly increased in *H*. *schachtii*-infected *A*. *thaliana* roots at 7 dpi (Figure 10) [23].

An elevated level of NO in tissues attacked by pathogens, especially within a short period of time after infection (in our case, 1–3 dpi), may promote the establishment of plant responses to biotic stress [43]. As we wrote above, at a later stage of the infection (7–15 dpi), to control the amount of NO at a level that would not threaten the functioning of the plant under infection conditions, the infected plant can activate specific tuning mechanisms to such stress conditions. We suggested that, at 7 dpi, GSNOR had a significant role in the catabolism of NO, while at 15 dpi, when the activity of this enzyme was impaired, we noticed that the non-symbiotic hemoglobin 1 (Hb1) gene transcript level was increased. Considering the NADPH-dependent dioxygenase activity of Hb1 that catabolizes NO to nitrate [18], our finding can indicate the participation of Hb1 in the scavenging of NO. It could also be confirmed by our recently published results, which showed the elevated content of nitrate ions in infected roots at 15 dpi [23].

## 5. Conclusions

The presented results support the presumption that the metabolism of RNS is an important biochemical player during plant interaction with cyst nematode (Figure 10). For the first time, the participation of NO and ONOO^−^ in plant responses against cyst nematodes is described. What is more, the obtained results suggest that GSNOR and non-symbiotic hemoglobin 1 can take part in controlling the bioactivity of NO in cyst nematode-infected plant roots. Therefore, our results supplement the knowledge gaps regarding the role of NO in the plant fight against cyst nematodes.

## Supplementary Materials

The following are available online at https://www.mdpi.com/2076-3921/9/9/795/s1, Figure S1: Negative control reactions for nitric oxide (a,c,e,g,i) and peroxynitrite (b,d,f,h) and detection with 4-amino-5-methylamino-20,70-difluorofluorescein diacetate (DAF-FM DA) and 3′-(*p*-aminophenyl) fluorescein (APF), respectively. In negative control samples, fluorescent probes were omitted. Abbreviations: N—nematode, S—syncytium, VC—vascular cylinder. Scale bars: 20 µm (a–e) and 100 µm (f–j); Figure S2: Negative controls for nitrosoglutathione reductase immunogold labelling. In negative control samples, primary antibody was omitted. Abbreviations: CW—cell wall, ER—endoplasmic reticulum, GA—Golgi apparatus, M—mitochondrion, Nu—nucleus, Pl—plastid, V—vacuole. Scale bars: 0.5 µm, original Images for Blots, original image for RNA agarose gel.
