# Supplementary material for: Cyst Nematode Infection Elicits Alteration in the Level of Reactive Nitrogen Species, Protein S-Nitrosylation and Nitration, and Nitrosoglutathione Reductase in Arabidopsis thaliana Roots
Source: Antioxidants (Basel). 2020 Aug 26;9(9):795. doi: 10.3390/antiox9090795 (PMC7555039; doi:10.3390/antiox9090795)
Supplement: Supplementary file 1 [file antioxidants-09-00795-s001.pdf]

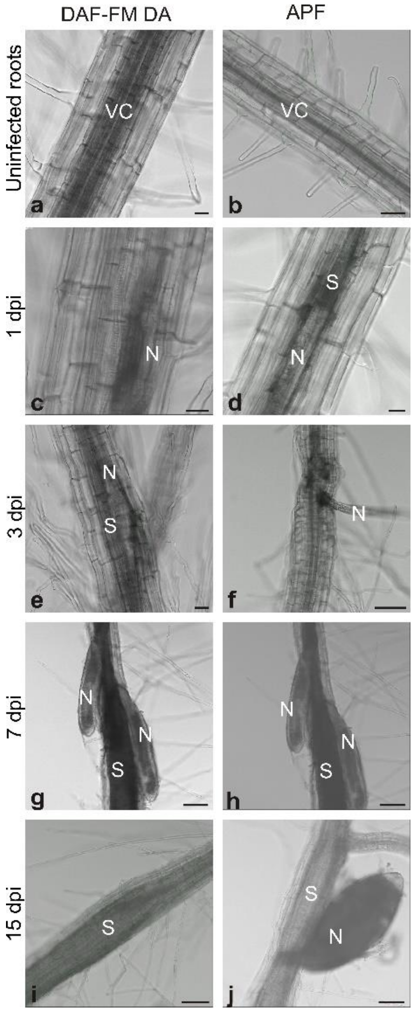

Figure S1. Negative control reactions for nitric oxide (**a, c, e, g, i**) and peroxynitrite (**b, d, f, h, j**) detection with 4-amino-5-methylamino-20,70-difluorofluorescein diacetate (DAF-FM DA) and 3′-(*p*-aminophenyl) fluorescein (APF), respectively. In negative control samples, fluorescent probes were omitted. Abbreviations: N- nematode, S- syncytium, VC- vascular cylinder. Scale bars: 20 μm (**a-e**) and 100 μm (**f-j**).

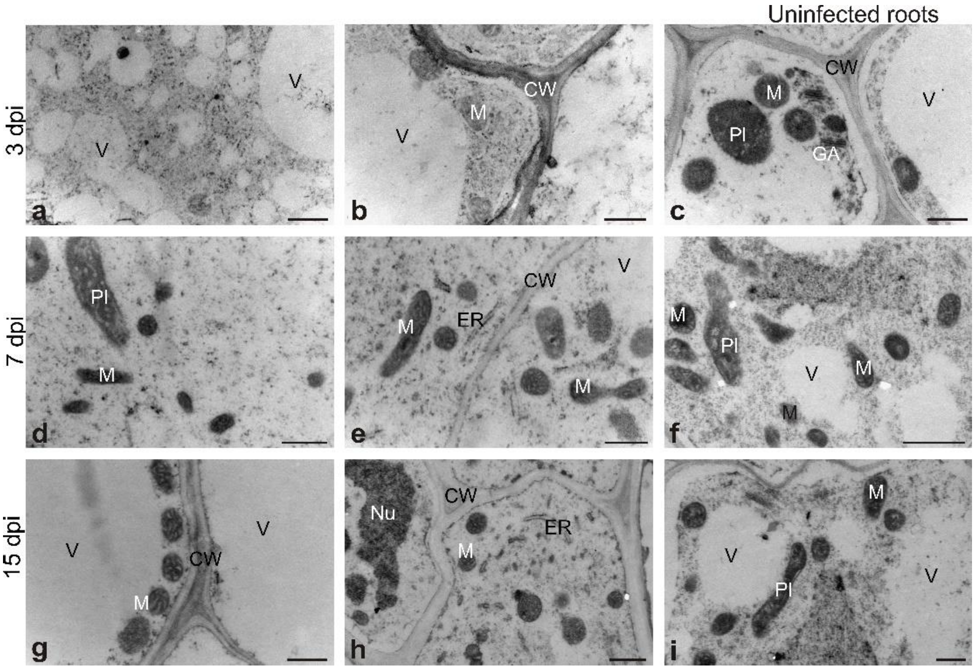

Figure S2. Negative controls for nitrosoglutathione reductase immunogold labelling. In negative control samples, primary antibodies were omitted. Abbreviations: CW- cell wall, ER- endoplasmic reticulum, GA- Golgi apparatus, M- mitochondrion, Nu- nucleus, Pl- plastid, V- vacuole. Scale bars: 0.5 μm.
